# Nitrous oxide and methane in a changing Arctic Ocean

**DOI:** 10.1007/s13280-021-01633-8

**Published:** 2021-10-10

**Authors:** Andrew P. Rees, Hermann W. Bange, Damian L. Arévalo-Martínez, Yuri Artioli, Dawn M. Ashby, Ian Brown, Hanna I. Campen, Darren R. Clark, Vassilis Kitidis, Gennadi Lessin, Glen A. Tarran, Carol Turley

**Affiliations:** 1 Plymouth Marine Laboratory, Prospect Place, The Hoe, Plymouth, PL1 3DH UK; 2 GEOMAR Helmholtz-Zentrum Für Ozeanforschung Kiel, Chemische Ozeanographie, Düsternbrooker Weg 20, 24105 Kiel, Germany

**Keywords:** Arctic Ocean, Environmental change, Ice retreat, Methane, Nitrous oxide, Ocean acidification, Warming

## Abstract

Human activities are changing the Arctic environment at an unprecedented rate resulting in rapid warming, freshening, sea ice retreat and ocean acidification of the Arctic Ocean. Trace gases such as nitrous oxide (N_2_O) and methane (CH_4_) play important roles in both the atmospheric reactivity and radiative budget of the Arctic and thus have a high potential to influence the region’s climate. However, little is known about how these rapid physical and chemical changes will impact the emissions of major climate-relevant trace gases from the Arctic Ocean. The combined consequences of these stressors present a complex combination of environmental changes which might impact on trace gas production and their subsequent release to the Arctic atmosphere. Here we present our current understanding of nitrous oxide and methane cycling in the Arctic Ocean and its relevance for regional and global atmosphere and climate and offer our thoughts on how this might change over coming decades.

**Supplementary Information:**

The online version contains supplementary material available at 10.1007/s13280-021-01633-8.

## Background

The Earth’s polar regions are rapidly changing as a direct result of our altered climate. The recent IPCC special report on the oceans and the cryosphere (IPCC 2019) found that “The polar regions are losing ice, and their oceans are changing rapidly. The consequences of this polar transition extend to the whole planet, and are affecting people in multiple ways”. The changes that are being experienced are having and will continue to have an increasing effect on the biogeochemical processes which are fundamental to the functioning of marine ecosystems (Rees 2012). The Arctic Ocean (AO) is experiencing this climate driven modification of its environment faster than anywhere else on the globe and there is high confidence that this region will likely become practically sea ice–free during the seasonal sea ice minimum for the first time before 2050, the practically ice-free state is projected to occur more often and with higher greenhouse gas concentrations (Fox-Kemper et al. 2021). To add to the complexity of this change, the loss of ice will be accompanied by the combined effects of increasing temperatures (Huang et al. 2017) and ocean acidification (AMAP 2018). Each of these artefacts of change have the potential to disrupt the biological processes which control the production and consumption of the atmospherically important trace gases nitrous oxide (N_2_O) and methane (CH_4_), whilst some of these effects may also modify the physical exchange between ocean layers or between the ocean and atmosphere.

As both of these gases are strongly radiatively active and their release from the Arctic Ocean will be impacted by ongoing and projected environmental changes, there is a need to further our understanding of how this might alter regional and global climate and ultimately impact society. The project “Pathways and emissions of climate-relevant trace gases in a changing Arctic Ocean (PETRA)” was designed to address these issues. In this paper we set observational and experimental evidence from a PETRA research cruise to the Fram Strait in July 2018 on board RV Polarstern (PS114) within the context of previous measurements of N_2_O and CH_4_ made by ourselves and others over the pan-Arctic region to assess our current understanding and to consider likely scenarios of change of N_2_O and CH_4_ in a future Arctic Ocean.

## Nitrous oxide (N_2_O)

N_2_O is a long-lived atmospheric trace gas whose atmospheric mixing ratio is increasing at a mean rate of 0.85 ± 0.03 ppb yr^–1^ (Canadell et al. 2021). It is rated as the third most important greenhouse gas (GHG) in the troposphere (Butler and Montzka 2018; Canadell et al. 2021) with a global warming potential on a 100-year timescale of approximately 300 times that of CO_2_ (Ramaswamy et al. 2001; Etminan et al. 2016) and is involved in ozone (O_3_) depletion in the stratosphere (Ravishankara et al. 2009). Though the N_2_O concentration in most of the surface of the ocean is in close equilibrium with the atmosphere (Nevison et al. 1995), global emissions from the open ocean and coastal waters contribute 35–39% of the total natural sources of N_2_O (Tian et al. 2020) and there is a fine balance between the ocean acting as net producer or consumer of N_2_O. Environmental effects associated with a changing climate, which include rising temperatures, oxygen depletion and ocean acidification are quite likely to impact the level of this equilibrium (Bange et al. 2019).

Nitrous oxide is biologically produced through three processes: Denitrification is the anaerobic reduction of NO_3_^−^ to N_2_ which has N_2_O as an intermediate; nitrification involves the two stage aerobic oxidation of NH_4_^+^ through NO_2_^−^ to NO_3_^−^, where the release of N_2_O as a by-product is dependent on the ambient O_2_ concentration (Goreau et al. 1980; Löscher et al. 2012). In the third route, nitrifier-denitrification, N_2_O can be formed during the reduction of NO_2_^−^ via nitric oxide to N_2_O by ammonia oxidizing bacteria. The pathway by which N_2_O is produced by ammonia oxidizing archaea is not yet fully understood (Wu et al. 2020).

## Methane (CH_4_)

CH_4_ is the most abundant organic trace gas in the environment, it plays an important role in the Earth’s climate and as a result of anthropogenic activities its atmospheric mixing ratio has more than doubled since the preindustrial era (Etminan et al. 2016). CH_4_ acts to limit the tropospheric oxidative capacity and is the second most important greenhouse gas, with a global warming potential that exceeds CO_2_ by up to 32 times over a 100-year timescale (Etminan et al. 2016; Canadell et al. 2021), contributing approximately 20% of the radiative climate forcing for all GHGs. The world’s oceans are a natural source of CH_4_ but play only a minor role in its global atmospheric budget, the open ocean and coastal waters account for 7 to 12% of the total natural sources and approximately 4% of global emissions (Saunois et al. 2020). It is thought that the open ocean contributes only a minor proportion of atmospheric CH_4_ with coastal environments including estuaries thought to account for approximately 75% of the total marine source (Weber et al. 2019).

The origin of CH_4_ in marine waters is from a diverse range of sources which may be geological, including from hydrothermal vents, cold seeps, and CH_4_ clathrates, or microbial in origin. The source of CH_4_ in ocean waters continues to prove enigmatic. Traditional understanding suggests that microbial methanogenesis is an anaerobic process, which in oceanic waters is thought to occur either in oxygen deplete waters or in anoxic micro-environments that are associated with zooplankton guts and particulate material (Brooks et al. 1981; Bianchi et al. 1992; Marty et al. 1997). However, the enhanced CH_4_ surface saturations found far away from shelf areas are difficult to explain because conventional CH_4_ production via archaeal methanogenesis should not occur in the well-oxygenated surface waters. This “ocean methane paradox” has been explained in tropical and sub-tropical oligotrophic conditions by the decomposition of methylphosphonate in phosphorus starved conditions (Karl et al. 2008) whereas in Arctic waters an alternative in situ CH_4_ production has been proposed following the microbial cleavage of dimethylsulphoniopropionate (DMSP) (Damm et al. 2008, 2010, 2015). Indeed DMSP is often found in very high concentrations in the AO during sea ice algal blooms and sea ice brines, and is projected to increase following ongoing changes to the Arctic environment (Campen et al. 2021).

## Current understanding of N_2_O and CH_4_ in Arctic waters

**Fig. 1:**
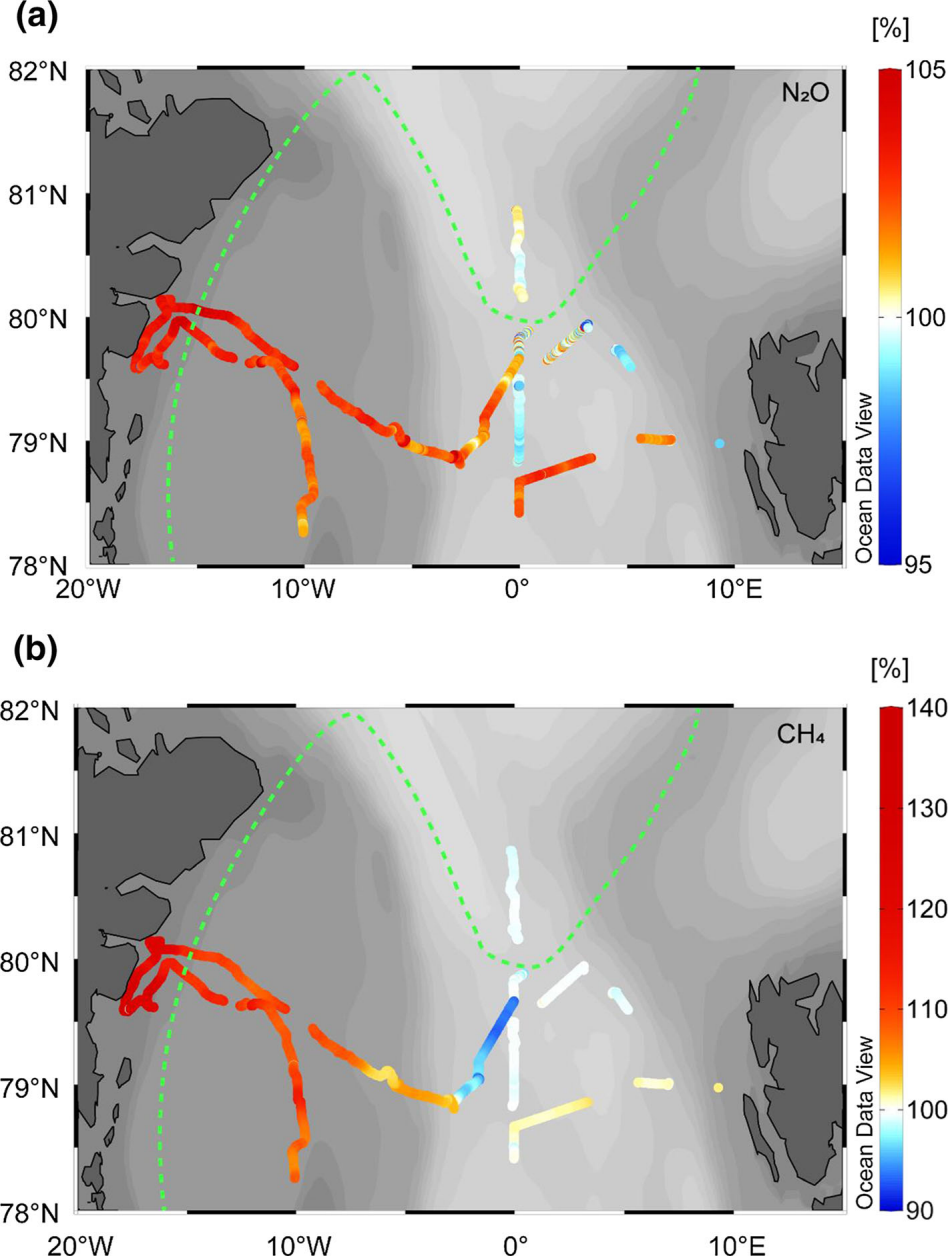
Distribution of (**a)** N_2_O and (**b)** CH_4_ saturation during research cruise PS114 onboard the RV *Polarstern* during July 2018. The green dotted line in each image represents the southern extent of sea ice at 90% cover (see Supplementary Material for method description)

Concentrations of dissolved N_2_O in the surface waters of Arctic shelf areas and central deep basins which exchange with the atmosphere are remarkably variable and can range from pronounced undersaturation to high supersaturation (Table 1). In general, ice-free surface waters appear to be undersaturated with N_2_O whereas ice-covered surface waters are supersaturated with N_2_O (Kitidis et al. 2010; Randall et al. 2012; Fenwick et al. 2017). For the North American AO it was suggested that high levels of N_2_O were largely associated with production in the shelf sediments of Chukchi and Bering Sea’s with subsequent advection eastwards (Fenwick et al. 2017). Supersaturations of N_2_O were also associated with the continental shelf for this region during the 7th Chinese National Arctic Research Expedition (Zhan et al. 2017, 2021) who also noted that offshore waters tended to be in equilibrium with the atmosphere. The Nordic Seas associated with the Greenland Basin and Fram Strait have been reported to be undersaturated in N_2_O (Rees et al. 2021) and act as a permanent sink to atmospheric N_2_O (Zhan et al. 2016). During research cruise PS114 onboard the German vessel RV *Polarstern* to the Fram Strait in July 2018 we measured concentrations and saturations of dissolved N_2_O and CH_4_ (Fig. 1) using an autonomous equilibrator headspace setup coupled to a trace gas cavity ringdown spectroscopy analyzer (Picarro Inc., USA; see Supplementary Material for methodology). The saturation of a dissolved gas indicates the relative amount of gas held relative to the value expected (100%) when the water body is in equilibrium with the overlying atmosphere. Supersaturated waters (> 100%) indicate a local source of the particular gas whereas a value of undersaturation (< 100%) indicates the potential for a sink to the atmosphere. In Fig. 1a, saturations of N_2_O can be seen to vary from a minimum of 95% in the southeast of the region close to Svalbard associated with the poleward West Spitsbergen Current and Atlantic influenced open-water. In the west of the region, higher levels of N_2_O, up to 107%, were found in shallower, ice-covered water close to Greenland and associated with the southerly flowing East Greenland Current. The heterogeneous distribution of N_2_O does not relate directly to surface ice-cover and maybe associated with the origin of source waters, though the higher concentrations to the west are likely the product of shelf sediment production and limited exchange with the atmosphere.

**Table 1:**
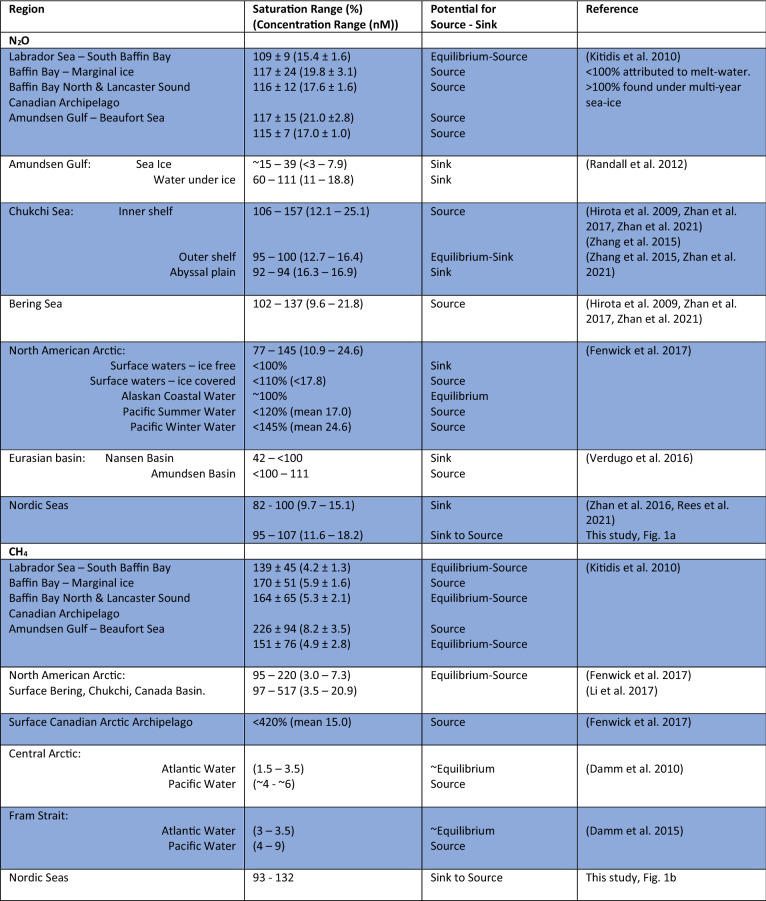
Exemplar N_2_O and CH_4_ publications from Arctic waters to indicate variability in regional source-sink characteristics for the two gases in the upper water column likely to exchange with the overlying atmosphere

At present, N_2_O emission estimates indicate that the overall net release of N_2_O from the AO to the atmosphere is comparably low (Fenwick et al. 2017). Microbial nitrification and/or denitrification in shelf sediments as well as water column nitrification have been proposed as the main N_2_O production pathways in the AO shelf areas and central deep basins (Kitidis et al. 2010; Verdugo et al. 2016; Fenwick et al. 2017), whereas loss processes include biological consumption (Verdugo et al. 2016; Rees et al. 2021) and physical advection (Zhan et al. 2016). Indications for the future release of N_2_O are conflicting. It was suggested that the ongoing decline of the Arctic sea ice cover may enhance future N_2_O emissions to the atmosphere (Kitidis et al. 2010) whilst in contrast, ongoing ocean acidification (AMAP 2018) has been shown to decrease N_2_O production in AO waters (Rees et al. 2016). The dominating N_2_O pathways and their dependence from changing environmental parameters/stressors (temperature, ocean acidification) and modifications of exchange across boundary layers need to be verified. Improved emission estimates of N_2_O to the atmosphere, as well as mechanistic understanding on how they might be affected by the above-mentioned perturbations is crucial since currently the uncertainties on the marine source of this gas to the atmosphere in the region are extremely high at approximately 100% (Yang et al. 2020).

Very high CH_4_ surface saturations have been observed in the shelf areas and the central deep basins of the AO (Table 1). Vast areas of the AO seafloor, particularly those associated with Siberian, Chukchi, and Beaufort Seas, is rich in permafrost (a potential substrate for methanogenesis) and CH_4_ hydrates (Chen et al. 2021). At present, the AO is a potentially important source of atmospheric CH_4_ (Kort et al. 2012) with sedimentary production via methanogenesis, dissociating gas hydrates and diffusion from geological dissolution providing the dominant source in shelf sea areas. Release of bubbles from sedimentary origin during the melting season represents a considerable, yet spatially constrained, source to the atmosphere (Thornton et al. 2020), though there is evidence to indicate that CH_4_ released at the sea bed may not reach the atmosphere, e.g. (Myhre et al. 2016). In Fig. 1b, CH_4_ saturations in the Fram Strait region during July 2018 can be seen to be highly variable. As with the distribution of N_2_O (Fig. 1a) and patterns of heterogeneity previously reported, minimum saturations (~ 93%) of CH_4_ were associated with Atlantic influenced ice-free waters and the highest observed (132%) were found in ice-covered areas close to the Greenland coast suggesting a strong source of CH_4_ to the air for this region.

Future CH_4_ emissions from open ocean regions of the AO will largely be determined by aerobic CH_4_ oxidation in the water column and ‘non-conventional’ microbial CH_4_ production via DMSP (Damm et al. 2010, 2015). The increased supply of organic matter from rivers and permafrost thaw may further enhance microbial methanogenesis. The effect of future environmental stressors such as warming and pH on aerobic CH_4_ oxidation is largely unknown (James et al. 2016).

## Evidence for potential change

### Impact of warming

Warming conditions are likely to impart direct and in-direct effects on the processes controlling production and consumption of both gases. Whereas the solubility of gases is governed by well constrained laws of physics under the control of temperature and salinity, so that warmer waters hold less gas than cold ones, the composition of microbial communities and their ecological function of changing systems is much less predictable. Warming would further enhance microbial methanogenesis with a 2° increase in temperature resulting in a 25–200% increase in methanogenesis (Bange et al. 1998). Finally, sea ice loss as a result of warming may result in shorter residence time for CH_4_ and N_2_O in the water and thereby evasion to the atmosphere rather than in-water microbial processing (sea ice is currently considered as a semi-permeable barrier to air-sea exchange) (Kitidis et al. 2010). Increasing seawater temperatures lower the solubility of CH_4_ in seawater and allow a shallowing of the CH_4_ hydrate stability zone. Therefore, a small increase in seawater temperature could potentially lead to hydrate dissolution and the subsequent release of CH_4_ from the AO to the atmosphere (Kitidis 2009).

There is some confidence that phytoplankton primary productivity is likely to increase as ice-cover retreats (Lannuzel et al. 2020) which will increase the sediment load of organic material that is potentially used in remineralisation processes in the generation of both N_2_O and CH_4_. Increases in primary production can be attributed to the interplay between two factors: first, the increased spatial and temporal extent of open waters, and second, the enhanced nutrient input brought about by mixing, upwelling and lateral advection, all of which is fostered by increased inflow from subpolar seas (the so-called Atlantification of Arctic waters) and more frequent storm events (Polyakov et al. 2017).

As ocean temperatures rise, CH_4_ hydrates may become unstable releasing vast quantities of CH_4_ to the atmosphere which in turn may lead to further temperature increase and hydrate de-stabilisation according to the “clathrate gun” hypothesis (Kennett et al. 2003).

Although CH_4_ solubility will decrease with increasing temperature, methanotrophy will also increase (Yvon-Durocher et al. 2014). Methanotrophy is the dominant oceanic CH_4_ sink and is a first order process with respect to CH_4_ concentration and inversely related to its turnover time. This suggests that an enhanced sedimentary release, potentially caused by warming triggered gas hydrate dissociation may result in shorter turnover times of dissolved CH_4_ in the water column (James et al. 2016). However, direct ebullition from dissociating gas hydrates or thawing permafrost may result in substantial emission of CH_4_ to the atmosphere as bubbles rise faster than they are consumed by microbes (Shakhova et al. 2010). Decreased sea ice would also reduce the extent of areas where bubbles released from the sediments are trapped. The transfer of these bubbles across the sea surface during the melting season might represent a spatially variable, yet considerable source of CH_4_ to the atmosphere (Zhou et al. 2014).

## Impact of ocean acidification

In the open oceans nitrification is the dominant mechanism for the production of N_2_O. A number of studies have shown that nitrification rate is inhibited by decreasing pH (Beman et al. 2010; Kitidis et al. 2011) but the impact on N_2_O appears equivocal. During experiments performed in Arctic and Antarctic waters (Rees et al. 2016) showed that whilst the microbial community of ammonia oxidising archaea (the dominant nitrifying organisms) seemed unaffected by changing pH, the production of N_2_O decreased at all stations by between 2.4 and 44% when pH was reduced at between values of 0.06 and 0.4 pH units. The reduction in N_2_O yield from nitrification was directly related to a decrease of between 28 and 67% in available NH_3_ as a result of the pH driven shift in the NH_3_:NH_4_^+^ equilibrium. In the subarctic western North Pacific (Breider et al. 2019) found that decreasing the pH during experimental manipulations acted to significantly increase N_2_O production, whilst rates of nitrification either remained stable or decreased, indicating a de-coupling of the two processes. The differences between the findings of Rees et al (2016) and Breider et al (2019) maybe attributable to regionally associated differences in N_2_O production pathways, or to the relative sensitivities of microbial communities found in the two regions.

**Fig. 2:**
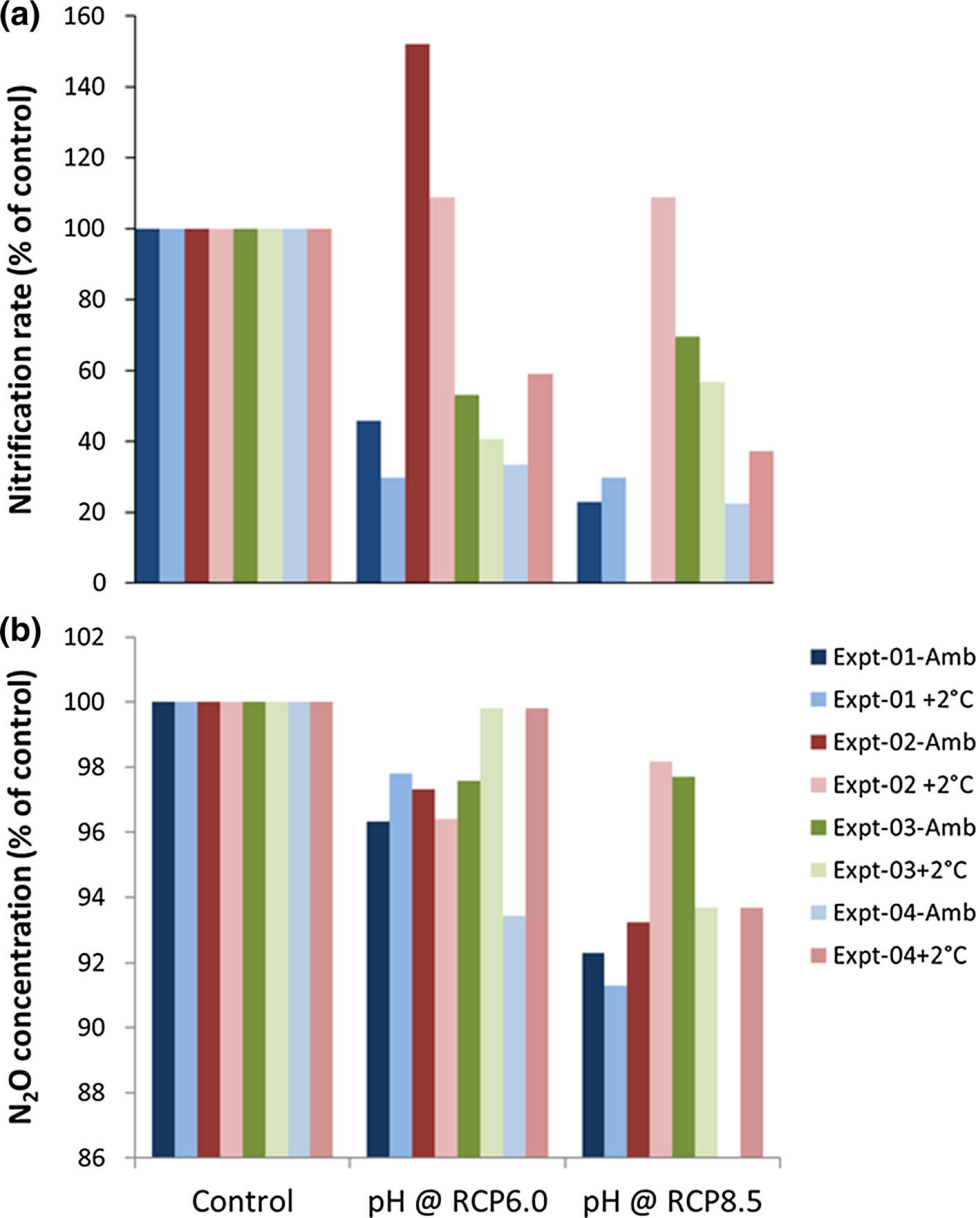
The impact of decreasing ocean pH and increasing temperature by 2 °C on (**a)** the rate of nitrification and (**b)** N_2_O concentration at four positions in the Fram Straits region of the AO during research cruise PS114 in July 2018 (see Supplementary Material for method description)

During the current project we performed four experiments during PS114 in the Fram Strait region similar to those described in Rees et al (2016). These were conducted to examine the impact of ocean acidification in isolation and in combination with warming of 2 °C (see Supplementary Material for methodology). Initial findings can be seen in Fig. 2. It would appear that there is a decrease in both N_2_O concentration and in nitrification rate with changes of pH which were made to match future conditions indicated by representative concentration pathway (RCP) of 6.0 and 8.5. Whilst there is some inherent variability in the responses, it would appear that there is no obvious response of N_2_O production or nitrification rate to warming of 2 °C.

To date we are unaware of experimental evidence to suggest that either methanogenesis or methanotrophy show any sensitivity to changing conditions of ocean acidification. A limited number of experiments that we have performed have all indicated that both CH_4_ production and consumption processes are likely to prove resilient to ocean acidification in the AO.

## Ecosystem modelling

The incorporation of up-to-date knowledge of N_2_O and CH_4_ dynamics in the marine environment into coupled physical-biogeochemical models is important to (a) expand our understanding and to test hypotheses related to the dynamics of these gases, (b) to explain spatial and temporal distribution patterns, and (c) to predict future change under the impact of multiple stressors. However there is currently insufficient understanding of the production and consumption pathways of these gases and their environmental controls which remains a limiting factor for their wider inclusion into process-based model studies, both in the AO and elsewhere.

A range of model formulations of N_2_O dynamics based on observational data have been developed in recent years and applied at a global scale, e.g. (Martinez-Rey et al. 2015; Ji et al. 2018) thus covering, but not focusing on, the AO region. However, implementation of regional-scale models of N_2_O is hindered by the scarcity of observational data and incompleteness in understanding of various pathways and their response to stressors, especially within high-gradient environments.

The issue is even more apparent for CH_4_, as many unknowns related to its production and consumption pathways still limit the rare modelling efforts to sensitivity studies, e.g. (Wåhlström and Meier 2014) who focused on the Laptev Sea region. Better understanding of the controls over the CH_4_ pathways is critical to constrain process-based models: for instance, the reported range of oceanic aerobic CH_4_ oxidation rates spans several orders of magnitude. In the AO the uncertainties in biological pathways are further augmented by a range of shelf sea processes related to permafrost thawing and CH_4_ hydrate dissolution, that all require advancements in understanding and consideration in the models.

The implementation of sophisticated process-based models which are able to project future emissions of N_2_O and CH_4_ under the influence of multiple stressors is severely hampered by sparse sampling (Weber et al. 2019) and lack of experimental evidence to advance statistically sound mechanistic understanding of the controlling processes. There is real need for an increased capacity of measurements such as those reported in this study to further develop this on both regional and global scales.

## Social and policy implications

N_2_O and CH_4_ contribute significantly to climate change. They are relevant to the United Nations Framework Convention on Climate Change (UNFCCC), the primary international, intergovernmental forum for negotiating the global response to climate change. The ultimate objective of the UNFCCC is to stabilize GHG concentrations "at a level that would prevent dangerous anthropogenic (human induced) interference with the climate system" (UNFCCC 1992). The goal of the Paris Agreement (UN 2015), a legally binding international treaty on climate change, adopted by 196 Parties at UNFCCC COP 21 in Paris in 2015, is “to limit global warming to well below 2, preferably to 1.5 °C, compared to pre-industrial levels”. To achieve this long-term temperature goal, countries aim to reach global peaking of GHG emissions as soon as possible to realize a climate neutral world by mid-century.

UN Member States adopted 17 Sustainable Development Goals (SDGs), as part of the 2030 Agenda for Sustainable Development (United Nations 2015), a global partnership for sustainable improvement of human lives whilst protecting the environment, including oceans, and tackling climate change. The oceanic production of N_2_O and CH_4_ addressed in this study is relevant to climate change goal (SDG13), to "Take urgent action to combat climate change and its impacts".

**Fig. 3:**
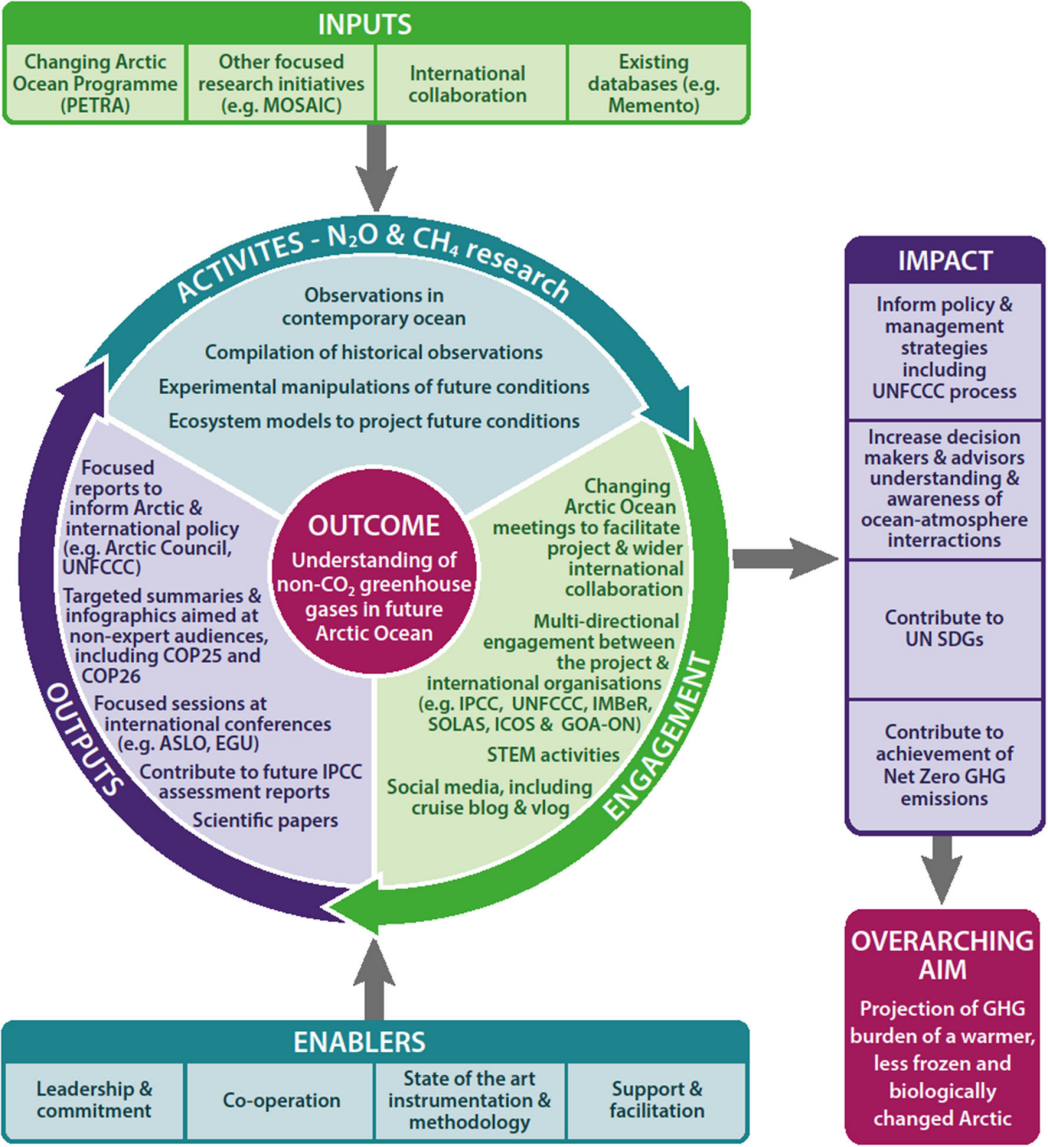
Theory of Change visualisation of social and policy implications associated with the PETRA project investigations of N_2_O and CH_4_ in a changing AO

The generation of impact is integral to PETRA (Fig. 3). Data generated will be managed and archived at our local oceanographic data centres (British Oceanographic Data Centre for UK and PANGEA for Germany) according to our data management plan. To ensure greater visibility and access, CO_2_ data collected during fieldwork expeditions in 2018 and 2019 have been submitted to the Surface Ocean CO_2_ Atlas (SOCAT) and included in the annual Global Carbon Project budget (Friedlingstein et al. 2020) whilst N_2_O and CH_4_ data will be submitted to the marine methane and nitrous oxide database (MEMENTO) (https://memento.geomar.de/). Outputs will also be made available through engagement with the GOA-ON observational network.

“The polar regions are losing ice, and their oceans are changing rapidly. The consequences of this polar transition extend to the whole planet, and are affecting people in multiple ways” (IPCC 2019). Understanding the role of the ocean in the cycling and production of N_2_O and CH_4_ and how these may change in an ocean undergoing rapid and long-term change is therefore essential to the Convention and climate negotiations. The findings of our previous work (Rees et al. 2016) and the newer observations from PETRA indicate that ocean acidification has the potential to decrease N_2_O emissions by up to 0.8 Tg N yr^−1^ which is comparable to all current N_2_O production from fossil fuel combustion and industrial processes of 0.7 Tg N yr^−1^. There is some potential therefore for decreases in N_2_O release to the atmosphere to offer a negative feedback to global warming, though it is still too early to say as other contrasting (Breider et al. 2019) and compounding effects are still to be accounted for.

## Outlook

Whilst estimates of the global marine source of N_2_O and CH_4_ to the atmosphere have significantly improved (Wilson et al. 2020), a mechanistic understanding of the causes for the observed variability in sink-source dynamics and sea/ice-air gradients in the AO is missing. This degree of uncertainty is highlighted in Table 2, where current understanding of the processes controlling these gases in concert with ocean acidification and warming are presented. Reduced sea ice coverage in the AO and the adjacent subpolar regions with future warming will likely expose larger ocean surfaces to direct exchange with the atmosphere, increasing the overall source of N_2_O and CH_4_ (a positive feedback on GHG-driven warming). However, the sea air transfer depends strongly on the pre-existing gradients between both reservoirs. The balance is a complex product of several processes. During the freezing period, brine rejection leads to gas enrichment and density-driven fluxes towards the underlying water column, but also potentially supports temporary fluxes towards the atmosphere. In contrast, during melting, a dilution effect causes gas undersaturation with respect to atmospheric equilibrium together with a strong salinity driven stratification. This would result in a net ocean uptake unless a strong source (e.g. sedimentary CH_4_ release) or buoyancy-driven flow breaks the stratification causing deep mixing and upwelling. At present it is challenging to establish which process is dominant over the annual cycle since the cycling of N_2_O and CH_4_ within the sea ice is not well understood and local production can be masked by lateral advection. Moreover, it is not clear to what extent an ocean acidification driven change of N_2_O production would contribute to offset the expected increase in the emissions to the atmosphere, and whether any synergistic effects may arise.

**Table 2:** Sources and sinks of N_2_O and CH_4_ in the AO, the expected effect of ocean warming (and associated melting) and acidification, as well as the level of uncertainties in the current state of knowledge

Processes	Source/sink	Estimated overall effect^a^		Level of uncertainty
		Warming	Ocean acidification	
N_2_O				
Nitrification	Source	↑	↓	Medium
Denitrification	Source/sink	↑	?	Medium
Air-sea exchange	Source/sink	↑	↓	High
Within-ice cycling & fluxes	Source/sink	?	?	Very high
CH_4_				
Methanogenesis	Source	↑	?	Medium
Methanotrophy	Sink	↑	?	Medium
Aerobic oxidation	Sink	↑	?	High
Air-sea exchange	Source/sink	↑	?	High
Within-ice cycling & fluxes	Source/sink	?	?	Very high

^a^↑ increase, ↓ decrease, ? unknown

Resolving gradients across the sea–ice–air interfaces and their spatial and temporal variability requires a combination of dedicated, multidisciplinary surveys, time series observations and the use of novel methods and autonomous platforms suitable for different ice conditions, e.g. (Bange et al. 2019; Lee et al. 2019). Studies investigating dynamics of N_2_O and CH_4_ within sea ice are scarce and therefore should be addressed in future joint projects. Recent observations from the MOSAiC expedition (https://mosaic-expedition.org/) and the Synoptic Arctic Survey (https://synopticarcticsurvey.w.uib.no/) are expected to provide important contributions towards a better understanding of the cycling and emissions of N_2_O and CH_4_ in the AO. Overall, strengthening observational capabilities in the AO will reduce the current emission uncertainties and thereby improve our projections of future GHG emission trends within the context of global coupled models.

## Supplementary Information

Below is the link to the electronic supplementary material.

Supplementary file1 (PDF 224 kb)
