# Supplementary material for: Nitrous oxide and methane in a changing Arctic Ocean
Source: Ambio. 2021 Oct 10;51(2):398–410. doi: 10.1007/s13280-021-01633-8 (PMC8692636; doi:10.1007/s13280-021-01633-8)
Supplement: Supplementary file 1 — Supplementary file1 (PDF 224 kb) [file 13280_2021_1633_MOESM1_ESM.pdf]

Ambio

Supplementary Information

This supplementary information has not been peer reviewed.

Title: **Nitrous oxide (N_2O) and methane (CH_4) in a changing Arctic Ocean**

Nitrous oxide (N_2O) and methane (CH_4) in a changing Arctic Ocean

Methods

Research cruise PS114 took place on the German research vessel RV Polarstern between 10 July 2018 and 3 August 2018 (Supplementary Fig. 1).

Continuous surface measurements of dissolved N_2O and CH_4 in seawater were carried out by means of an autonomous equilibrator headspace setup coupled to a trace gas cavity ringdown spectroscopy analyzer. Seawater was drawn from approximately 11 m depth into the system by using the ship's continuous supply. Control measurements and calibration procedures were performed every 24 h by means of two standard gas mixtures (Deuste Steininger GmbH, Germany) bracketing the expected concentrations in this area. The principle of analysis involves equilibrating air with continuous running seawater and circulating the air phase through the detector and returning in a closed circulation loop. N_2O and CH_4 measurements were carried out by a Picarro cavity ring down spectrometer G2508 (Picarro Research Inc., USA) which produces simultaneous measurements of molar fractions of N_2O and CH_4 as well as water vapour with precision at sub-ppb levels.

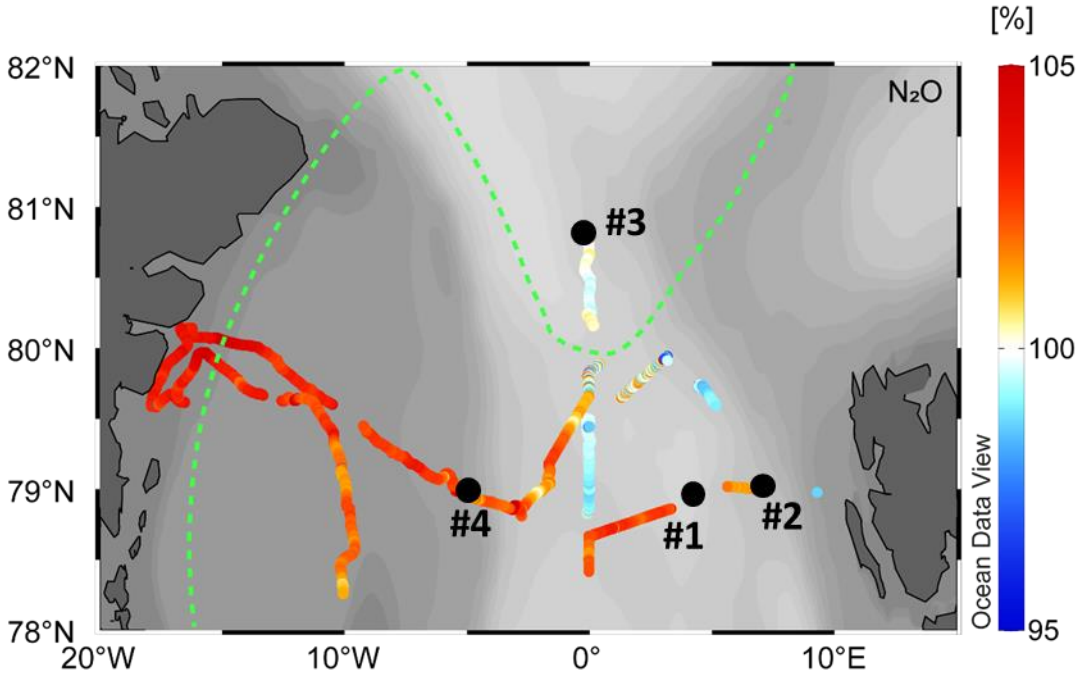

Supplementary Fig.1: Nitrous oxide saturation in near-surface waters of the Fram Strait indicated by filled coloured circles, the positions of which reflect the ship's track between 19th and 29th July 2018. Experimental stations (#1 to #4 are indicated by the filled black circles.

Incubation experiments were performed at four stations indicated in Supplementary Fig. 1 to assess the impact of ocean warming and ocean acidification on the production/consumption of N_2O and CH_4 and also on the rates of nitrification. Incubations were carried out at ambient temperature and ambient plus 2°C, pH was changed by adding 1M hydrochloric acid and 1M bicarbonate solutions, similar to (Rees et al. 2016). For all experiments, large volumes of water were drawn from the CTD

rosette and after amendment were incubated in temperature-controlled experimental enclosures for up to 96 h. Time series measurements of N_2O and CH_4 were made following addition of mercuric chloride after 12 hours, 24 hours, 48 hours and 96 hours. 1 litre seawater samples were equilibrated with compressed air and headspace analysis performed onboard using flame ionisation detection-gas chromatography and electron capture detection-gas chromatography for CH_4 and N_2O respectively (Upstill-Goddard et al. 1996). Rates of nitrification were made after 46 hours of incubation following the inhibition of ammonium oxidation by allylthiourea and nitrite oxidation by sodium chlorate. Ammonium oxidation rates are determined as the NO_2^- accumulation rates in $NaClO_3$-treated sediment compared to ATU treated samples (Kitidis et al. 2011).

References

Kitidis, V., B. Laverock, L.C. McNeill, A. Beesley, D. Cummings, K. Tait, M.A. Osborn, and S. Widdicombe. 2011. Impact of ocean acidification on benthic and water column ammonia oxidation. *Geophys. Res. Lett.* 38: L21603.

Rees, A.P., I.J. Brown, A. Jayakumar, and B.B. Ward. 2016. The inhibition of N_2O production by ocean acidification in cold temperate and polar waters. *Deep-Sea Research Part Ii-Topical Studies in Oceanography* 127: 93-101. https://doi.org/10.1016/j.dsr2.2015.12.006

Upstill-Goddard, R.C., A.P. Rees, and N.J.P. Owens. 1996. Simultaneous high-precision measurements of methane and nitrous oxide in water and seawater by single phase equilibration gas chromatography. *Deep-Sea Research Part I-Oceanographic Research Papers* 43: 1669-1682. https://doi.org/10.1016/s0967-0637(96)00074-x
